# Q RadFusion: Hybrid Quantum Classical Radiogenomic Framework for Breast Cancer Diagnosis

**DOI:** 10.21203/rs.3.rs-7657076/v1

**Published:** 2025-09-23

**Authors:** Padmaja C, Sivaneasan Bala Krishnan S., Ramacharan S, Prasun Chakrabarti

**Affiliations:** G Narayanamma Institute of Technology and Science; Singapore Institute of Technology; G Narayanamma Institute of Technology and Science; Singapore Institute of Technology

**Keywords:** Radiogenomics, Breast Cancer, CBIS-DDSM, TCGA-BRCA, Quantum AI, QAOA, VQC, Multimodal Fusion

## Abstract

**Background and Purpose:**

Breast cancer remains the most common cancer in women worldwide, with early and accurate diagnosis critical for patient survival. Radiogenomics integrates imaging phenotypes with genomic profiles, offering a pathway to precision diagnostics. However, existing classical machine learning models often struggle with the high dimensionality and heterogeneity of multimodal data, leading to issues in calibration and reproducibility. This study presents Q RadFusion, a hybrid quantum-classical framework designed to enhance breast cancer diagnosis by fusing mammography and genomics data.

**Methods:**

Q RadFusion was implemented on two publicly available datasets: CBIS-DDSM (2,600 curated mammography cases, TCIA) and TCGA-BRCA (1,000 genomic profiles, GDC). Imaging preprocessing included bias-field correction, segmentation, and harmonization, while genomic data underwent normalization and imputation. Feature selection was performed using the Quantum Approximate Optimization Algorithm (QAOA), and features were mapped into a quantum Hilbert space using Variational Quantum Circuits (VQC). For multimodal fusion, ResNet encoded mammography features, and a Transformer encoded genomic features. Patient-level and site-held-out splits were used for evaluation.

**Results:**

Q RadFusion achieved an AUC of 0.96 and accuracy of 94%, outperforming baselines including CNN-LSTM, ResNet + XGBoost, and multimodal Transformers. Ablation studies confirmed the contribution of quantum components, with optimal performance observed at circuit depth L=6, qubits Q=10, and QAOA layers p=3. The model also demonstrated improved calibration and ~ 80% fewer parameters compared to deep fusion networks.

**Conclusion:**

Q RadFusion demonstrates that hybrid quantum–classical radiogenomic integration can deliver accurate, reproducible, and clinically meaningful diagnostic support for breast cancer, with strong potential for future clinical translation.

## 1. Introduction

### 1.1. Background on Breast Cancer and Radiogenomics

Breast cancer is the most prevalent cancer among women worldwide and continues to be a leading cause of mortality despite significant advances in screening and treatment. It is estimated that over 2.3 million women are diagnosed annually, with the death rates being the highest in those areas where primary access to advanced diagnostics is unavailable [1]. Mammography, biopsy, and histopathology are the conventional methods of diagnosis used in clinical practice. Their performance is, however, limited by tumor heterogeneity, variability in image interpretation, and the lack of capacity to capture the molecular complexity of the disease. This mismatch between imaging-based imaging and genomic-level comprehension highlights why integrative methods that better capture the biology of the tumor are essential [2].

Radiogenomics has emerged to become an influential paradigm of bridging this divide between imaging phenotypes and genomic profiles. Radiomics features captured in mammography, including the morphological, textural, and intensity-based feature sets, can be related to genomic alteration, including BRCA1/2 mutations, copy number variation, and RNA expression profiles[3]. It is possible to apply these associations and forecast tumor subtype, prognosis, and response to treatment through non-invasive tumor subtype prediction radiogenomic techniques. Taking a case in point, a study on the association of mammographic phenotypes with HER2 or estrogen receptor status has demonstrated that imaging biomarkers can be utilized to target clinical decisions without having to conduct an invasive procedure [4,5].

Beyond drawing on these datasets, there has been a marked increase in the availability of large-scale, publicly available, readily usable datasets that have allowed researchers to advance radiogenomic research. The collection of CBIS-DDSM, which is curated by the Cancer Imaging Archive, includes over 2,600 labeled cases of mammography and includes a suitable foundation of imaging [6]. The TCGA-BRCA data subset is available on the Genomic Data Commons with approximately 1000 genomic profiles of breast cancer, including RNA-Seq and copy number data, in parallel [7]. Collectively, such resources offer a baseline to integrative platforms like Q RadFusion, which are designed to merge imaging and omics data to build more effective early diagnosis and precision oncology.

### 1.2. Challenges in High-Dimensional Multimodal Data

Radiogenomics holds great promise in the transformation of breast cancer diagnosis, but its practical application in our daily lives has its share of challenges. The main concerns are the high dimensionality of the multimodal data. A single mammogram alone may contain thousands of radiomic features, whereas a single genomic analysis may contain tens of thousands of variables per patient [8]. When these are combined, they end up with a thoroughly vast feature space that is out of proportion compared to the available sample sizes. Such an imbalance is commonly termed the curse of dimensionality, which makes overfitting more likely to occur and restricts the ability to generalize diagnostic models.

Intermodal differences pose other challenges as well. Genetic and imaging data intrinsically differ in scale, structure, and statistical distribution, making it complex to formulate harmonization strategies. Batch effects and noise, in addition to variations that imaging equipment or sequencing platforms introduce, cause inconsistencies, making it difficult to compare studies. Classical machine learning and deep learning models have proved ineffective at capturing nonlinear and hierarchical relationships among the various modalities and computationally demanding, with a large parameter count, making them unstable in low-sample settings.

One more severe problem is calibration and interpretability. Although the reported accuracies of many deep learning models are high, these models can have poor estimates of the probability of classification error that prevent their accurate clinical decision-making. Moreover, the ambiguity of most of the available models creates distrust among clinicians and inhibits application in practice. To overcome such limitations, new frameworks are necessary that could minimize the dimensionality and enable effective alignment of multimodal features and provide calibrated and reproducible results. Such difficulties are the intellectual justification for creating hybrid quantum-classical techniques like Q RadFusion that utilize quantum feature spaces and deep classical encoders to bypass the weaknesses of traditional radiogenomics.

### 1.3. Role of Quantum AI in Healthcare

The potential of quantum artificial intelligence within data-driven healthcare is becoming acknowledged. In contrast to regular algorithms, however, quantum systems use the phenomena of superposition and entanglement to represent information in higher-dimensional Hilbert spaces. This enables the more efficient modeling of high-dimensional and complex correlations of biomedical data. In the setting of breast cancer radiogenomics, quantum feature encoding and variational quantum circuits have the potential to detect the subtle interplay of non-linear effects between imaging biomarkers and genomic alterations that would be difficult to resolve using classical methods [9].

Recent developments have also illustrated the promise of quantum-enhanced classifiers and quantum kernels in medical imaging tasks, including histopathology and using MRIs to detect conditions [10]. The experiments done by these papers are still at the proof-of-concept level; however, they show the superiority of quantum feature space in the performance of classification tasks and robustness against the necessity of a giant training set. Importantly, the combination of quantum methods with classical deep learning encoders, such as ResNet for imaging and Transformers for genomic data, aligns well with the noisy intermediate-scale quantum era, where fully fault-tolerant quantum computers are not yet available [11]. Hybrid architectures thus provide a practical path toward clinical applications by blending the strengths of both paradigms.

### 1.4. Literature Gap and Problem Statement

Despite substantial progress in radiogenomics and artificial intelligence, significant gaps remain that hinder translation into routine clinical practice. Existing multimodal studies often rely exclusively on classical machine learning or deep learning methods that are not optimized for the extreme dimensionality of imaging and genomic data. While these models can report competitive accuracy, they frequently lack calibration, reproducibility, and interpretability. This limits their ability to support clinical decisions where reliability is critical.

Furthermore, current quantum machine learning research in healthcare has largely focused on single-modality imaging or theoretical demonstrations, with very few applications addressing multimodal integration. To date, there is limited evidence of reproducible hybrid quantum classical radiogenomic frameworks that combine mammography and genomic data to deliver clinically meaningful outcomes. The absence of such integrative approaches leaves a gap between technological potential and clinical utility.

The problem addressed in this study is therefore the development of a reproducible, efficient, and interpretable framework that integrates CBIS-DDSM mammography with TCGA-BRCA genomic profiles through hybrid quantum classical methods. The aim is to demonstrate superior accuracy, calibration, and parameter efficiency compared with established baselines, while ensuring transparency and reproducibility through publicly accessible datasets and pseudocode.

### 1.5. Study Objectives and Contribution

This study proposes Q RadFusion, a hybrid quantum-classical framework for breast cancer radiogenomics. The objectives are fourfold. First, to design a reproducible diagnostic pipeline that integrates CBIS-DDSM mammography with TCGA-BRCA genomic data. Second, to implement QAOA for feature selection and VQC-based quantum feature encoding. Third, to combine quantum layers with deep encoders, specifically ResNet for imaging and Transformers for genomics, to achieve robust multimodal fusion. Fourth, to evaluate model performance through patient-level and site-held-out validation while ensuring calibration and efficiency.

The contributions of this work are the introduction of the first reproducible quantum-classical radiogenomic framework applied to breast cancer, the demonstration of improved diagnostic accuracy and calibration, and the provision of a clinically relevant model grounded in publicly available datasets.

## 2. Related Work

### 2.1. Radiogenomics in Breast Cancer Diagnosis

Radiogenomics represents a rapidly growing area within precision oncology that seeks to establish associations between imaging phenotypes and genomic profiles, thereby enabling non-invasive insight into tumor biology. In breast cancer, where heterogeneity at both the morphological and molecular levels complicates diagnosis and treatment, radiogenomics offers an integrative approach to patient stratification and prognosis prediction [12].

Mammography remains the frontline screening tool for breast cancer detection, and large-scale resources such as the CBIS-DDSM dataset, hosted by The Cancer Imaging Archive, have facilitated the systematic extraction of radiomic features. These features, including textural, morphological, and statistical descriptors, have been linked to clinically significant genomic alterations. For example, studies have explored correlations between mammographic texture patterns and BRCA1/2 mutation status, while others have examined imaging associations with receptor subtypes such as estrogen receptor (ER) and HER2 [13]. Such findings suggest that imaging biomarkers may serve as non-invasive surrogates for molecular assays.

On the genomics side, the TCGA-BRCA cohort provides a comprehensive collection of multi-omics profiles, including RNA-Seq expression data and copy number variations, for over 1,000 breast cancer patients [6]. This dataset has enabled radiogenomic studies that correlate imaging-derived features with molecular subtypes and survival outcomes [14]. Integrative analyses across CBIS-DDSM and TCGA-BRCA demonstrate the feasibility of linking radiomic signatures with gene expression to enhance diagnostic precision.

Despite promising results, most prior work in breast cancer radiogenomics has employed classical statistical or machine learning approaches, which struggle with high-dimensional integration. These limitations underscore the need for advanced frameworks such as Q RadFusion, which combines quantum feature encoding with classical deep encoders to better capture the complex, non-linear relationships between imaging and genomic data.

### 2.2. Radiomics and Omics AI Frameworks

The combination of radiomics data and omics data has become an important solution for the future of breast cancer precision medicine. Radiomics characterizes tumor heterogeneity using high-throughput quantitative features that describe shape, intensity, and texture characteristics of a medical image, such as a mammography [15]. Omics data, including transcriptomics, genomics, and proteomics, capture the molecular mechanisms underlying tumor progression and therapeutic response. When integrated, these modalities provide complementary insights: radiomics reflects phenotypic heterogeneity, while omics identifies genomic and transcriptomic drivers of disease.

Public resources such as CBIS-DDSM and TCGA-BRCA have enabled multimodal research by providing paired imaging and genomic datasets. Studies using these datasets have shown that combining mammography-derived features with RNA-Seq and copy number variation profiles improves the prediction of molecular subtypes and patient outcomes compared with unimodal analyses [16]. Traditional machine learning models such as support vector machines and random forests were among the first applied to these tasks, often using handcrafted features selected via dimensionality reduction techniques.

More recent progress has shifted towards deep learning frameworks. Convolutional neural networks (CNNs) have been used to generate radiomic representations without user interaction or kernels, whereas sequential dependencies were learnt in genomic data using recurrent and Transformer-based networks [17]. Hybrid multimodal architectures that combine CNN-learnt image features with omics-learnt embeddings have been shown to provide better classification results [18]. Nevertheless, these frameworks are restrained by the problem of overfitting (small sample size in comparison with the number of features) and inadequate probability calibration and interpretability. A deficit of reproducible pipelines lowers clinical trust. These weaknesses point to the importance of new integrative techniques like Q RadFusion that address the issue of dimensionality by using QAOA-based feature selection and variational quantum circuits to help increase the reliability of multimodal fusion.

### 2.3. Quantum Machine Learning in Medical Imaging

Quantum machine learning (QML) holds promise as a sub-discipline with links to both quantum computing and artificial intelligence, introducing new strategies to the high-dimensional data challenges present in biomedical applications of AI [19]. Using quantum principles, including superposition and entanglement, QML models are able to process highly complex data into higher-dimensional feature spaces wherein non-linear relationships become easier to manage [20]. This is especially true of medical imaging, wherein very small changes in the texture and morphology commonly correlate with changes in the disease state but are typically hard to detect using classical techniques alone.

Medical applications of quantum-enhanced classifiers and quantum kernels include recent proof-of-concept studies: MRI-based tumor detection, histopathological image classification, and genomic sequence analysis [21]. These studies report competitive or superior performance compared with classical baselines, even when operating on limited training data. Hybrid models that combine quantum circuits with classical deep learning encoders have been especially promising, as they allow current noisy intermediate-scale quantum (NISQ) devices to contribute to clinical applications without requiring fully fault-tolerant quantum computers [22].

In the context of breast cancer radiogenomics, QML remains underexplored. Very few studies have attempted to integrate quantum circuits into multimodal frameworks that combine mammography and genomic data [12]. Existing research primarily focuses on single-modality classification or simulation-based experiments rather than clinically oriented, reproducible pipelines. This gap presents an opportunity for Q RadFusion, which is designed to combine quantum feature encoding, variational quantum circuits, and QAOA feature selection with deep learning encoders such as ResNet and Transformer. By doing so, Q RadFusion not only addresses the dimensionality and calibration challenges of radiogenomic integration but also positions quantum AI as a practical tool for advancing breast cancer diagnosis.

### 2.4. Hybrid Quantum Classical Diagnostic Models

The convergence of quantum computing and classical deep learning has given rise to hybrid quantum-classical diagnostic models, which aim to combine the strengths of both paradigms. Quantum algorithms provide efficient feature encoding and the ability to explore high-dimensional spaces, while classical models bring scalability, interpretability, and established infrastructure [23]. Together, these approaches form practical solutions well suited to the noisy intermediate-scale quantum era.

In medical imaging and genomics, hybrid models have been tested in tasks such as tumor classification, molecular subtyping, and biomarker discovery. For instance, variational quantum circuits have been integrated with convolutional neural networks to improve image-based tumor detection, while quantum kernel methods have been applied to genomic sequence analysis [24]. These studies show that quantum components can enhance feature representation and reduce dependence on large sample sizes.

Despite these encouraging results, most existing hybrid models remain at proof-of-concept levels, with limited validation on real-world datasets. In breast cancer, few attempts have been made to integrate both imaging and genomic modalities within a quantum-enhanced framework. This is significant because radiogenomics inherently requires multimodal fusion, where imaging phenotypes must be aligned with molecular alterations. Classical deep learning pipelines, although powerful, often suffer from poor calibration and interpretability, especially when trained on limited samples. Hybrid models that incorporate quantum feature selection and encoding offer a promising direction by addressing dimensionality, improving calibration, and delivering more reliable outputs [23]. Q RadFusion takes a step in advancing this area of research by providing a reproducible dataset-driven radiogenomics integration framework.

### 2.5. Gap Analysis and Novelty of Q RadFusion

Although the use of radiogenomics and artificial intelligence has been seen to have significant application in breast cancer diagnostics, the lack of reproducible hybrid frameworks has been a major loophole. Current radiogenomic experiments with CBIS-DDSM and TCGA-BRCA solely rely on conventional machine learning or deep learning, which have consistent limitations around dimensionality, calibration, and clinical/medical credibility. Similarly, quantum machine learning applications in healthcare remain incipient and are mostly single-modality-based, as opposed to internalized pipelines.

Q RadFusion fulfills these gaps by introducing a new hybrid quantum classical radiogenomic framework that combines mammography into CBIS-DDSM with genomics into TCGA-BRCA. Its novelty is the integration of the QAOA-based feature selection, quantum feature encoding with variational quantum circuits, the classical deep encoders, i.e., ResNet and Transformer, in multimodal fusion. By validating performance through patient-level and site-held-out splits, Q RadFusion demonstrates both accuracy and reproducibility. This positions it as the first comprehensive framework to bring quantum AI into practical, multimodal breast cancer diagnosis.

## 3. Materials and Methods

### 3.1. Datasets

This study employed two publicly available and widely recognized datasets that provide complementary imaging and genomic perspectives on breast cancer: the Curated Breast Imaging Subset of the Digital Database for Screening Mammography (CBIS-DDSM) and The Cancer Genome Atlas Breast Invasive Carcinoma (TCGA-BRCA) cohort.

The CBIS-DDSM dataset, hosted on The Cancer Imaging Archive (TCIA), consists of approximately 2,600 curated mammography cases [25]. It is a revised and standardized form of the original DDSM collection designed to ease studies in computer-aided diagnosis [16]. The data contains digitized film mammograms whose boundary shapes and other ground truth data are provided, including pathology-confirmed labels and lesion type (benign or malignant). BIS-DDSM has gained recognition as a widely used radiomics and machine learning dataset because of numerous reasons, such as being in a standardized format, extra curation, and metadata that encourage reproducible studies. The scale and quality of its annotation attribute it a special suitability in the extraction of radiomic features, which would include the texture, shape, and intensity descriptors, which can serve as a phenotypic characteristic of tumor biology. CGA-BRCA data available in the Genomic Data Commons (GDC) is a two-year genomic and transcriptomic dataset on 1,000 breast invasive carcinoma patients [7]. It contains RNA-Seq expression data, copy number variation (CNV) data, and homologous recombination deficiency (HRD) scores, along with clinical-correlated data of the patient, such as age, receptor status, and survival data. The information has been symbolized by a comprehensive molecular description and has gained immense popularity in the field of precision medicine research and cancer genomics. TCGA-BRCA can be used to capture the genomic alterations, signalling pathway defects, as well as the molecular subtypes that are vital in providing personalised care to breast cancer patients.

The integration of CBIS-DDSM and TCGA-BRCA can form a very powerful foundation of radiogenomic fusion. The imaging dataset contains the phenotypic descriptions of tumors, whereas the genomics dataset can include the molecular and transcriptomic information. They have been publicly available to guarantee reproducibility, ethical compliance, and transparency, and they do not require any additional ethical approval due to the complete anonymization of the data. This combination can be used to develop Q RadFusion: a hybrid quantum-classical tool to track mammographic features to genomic changes to enhance diagnostic precision and calibration.

### 3.2. Preprocessing Steps

Any imaging and genomic data must be preprocessed to be reliable and robust in radiogenomic integration. In this work, different yet synergistic pipelines were applied to the CBIS-DDSM mammography images and the TCGA-BRCA genomic data so that the two modalities were harmonized before the application of feature selection and multimodal fusion [25].

Regarding imaging data, preprocessing started with removing biases to solve intensity inhomogeneity in mammograms. This ensured that the variation introduced by the scanner or digitization artifacts was minimised, thus making its extracted radiomic features more consistent. Segmentation of the lesion was done depending upon the annotations that were given in the CBIS-DDSM dataset, whereby the regions of interest were isolated, i.e., the tumors were isolated. Radiomic feature extraction was then done to pick up the intensity, shape, and texture-based descriptors after the segmentation [6]. The acquisition protocols varied, so differences were considered by means of harmonization techniques, including z-score normalization and resampling to a given pixel spacing [8]. The harmonization minimized inter-sample variability and allowed the dataset to be used confidently in machine learning as well as quantum feature encoding.

To preprocess genomic data, normalization and imputation were used. Gene expression data obtained using RNA-Seq were normalized to enable a comparison within and between samples through a process like transcripts per million (TPM) or fragments per kilobase per million (FPKM). The data on copy number variation and HRD were preprocessed to match the standardized genomic annotations. Imputation methods, including k-nearest neighbors or expectation-maximization algorithms, were used to deal with missing values, which are prevalent in big-scale omics data [20]. Dimensionality reduction was conducted at this phase in order to eliminate low-variance features or features containing very little information, thus improving computing speed.

A combination of these preprocessing pipelines resulted in both the imaging and genomic modalities being ready to implement into the Q RadFusion system. The consistency in the imaging and genomic profiles due to harmonization and normalization of the imaging features and genomic profiles allowed quantum feature selection and multimodal fusion to be performed reproducibly and in a clinically relevant way.

### 3.3. Proposed Q RadFusion Framework

The proposed Q RadFusion framework will be an iterable hybrid quantum classical model that combines mammography radiomics of CBIS-DDSM with genozyme features of TCGA-BRCA. The framework takes advantage of quantum feature encoding, based on variational quantum circuits (VQC), quantum approximate optimization algorithm (QAOA), to perform feature selection, and classical deep encoders to fuse the multimodal data [26,27]. This section represents every element and gives mathematical formulations that act as the basis of the architecture.

#### 3.3.1. Quantum Feature Encoding

Quantum feature encoding offers the possibility to encode classic data into quantum states in high-dimensional Hilbert spaces to represent multimodal features richly. For an input feature vector x=(x1,x2,…,xn), angle encoding is applied:

$$\left[x\geq ⨂(i=1‥n)\left[\text{cos}\left(x_{i}/2\right)\left|0>+\text{sin}\left(x_{i}/2\right)\right|1>\right]\right]$$

Composing radiomics features with omics data will be done through an encoding that maps each feature to a quantum state, thus capturing nonlinearly more information than the classical treatment [26].

#### 3.3.2. Variational Quantum Circuit (VQC) Design

The encoded data are then operated by means of parameterized quantum circuits, in which unitary operations consist of variations between rotation and entanglement gates [27]. A general variational circuit is represented as:

|ψ(θ)≥U(θ)|

where U(θ) is the unitary operator parameterized by trainable variables theta θ [28]. Measurement outcomes are obtained by computing expectation values:

$$\left\langle O\right\rangle =\left\langle \psi \left(\theta \right)\left|O\right|\psi \left(\theta \right)\right\rangle ,$$

With O denoting the observable (e.g., Pauli-Z operators). These expectation values form the quantum-derived features used in downstream fusion.

#### 3.3.3. QAOA Feature Selection

Given the high dimensionality of multimodal features, QAOA is employed to optimize feature subsets. The QAOA algorithm alternates between a cost Hamiltonian $H_{C}$ and a mixing Hamiltonian $H_{M}$:

$$\left|\gamma ,\beta \geq \prod (j=1‥p)e^{\left(-i\beta_{j}H_{M}\right)}e^{\left(-i\gamma_{j}H_{C}\right)}\right|s>$$

Where γ,β gamma, are tunable parameters, p is the number of layers, and |s⟩ is the initial state. In this study, $H_{C}$ Encodes the objective of maximizing classification accuracy while penalizing redundant features. Feature subsets are selected by sampling measurement outcomes from the final QAOA state [29].

#### 3.3.4. Deep Encoders: ResNet and Transformer

In parallel to quantum modules, modality-specific encoders extract domain-relevant features. For imaging, a ResNet-based encoder processes segmented mammograms to generate radiomic embeddings [30]. Let the ResNet encoder be denoted by:

$$z_{\text{img}}=f_{\text{ResNet}}\left(I\right),$$

where I represents a preprocessed mammogram and $z_{\text{img}}$ is the learned image embedding.

For genomics, a Transformer encoder is applied to RNA-Seq and CNV vectors [31]. The self-attention mechanism computes contextual embeddings as:

$$\text{Attention}\left(Q,K,V\right)=\text{softmax}\left(\frac{QK^{T}}{\sqrt{d_{k}}}\right)V,$$

Where queries Q, keys K, and values V are derived from linear transformations of the genomic feature vectors. The Transformer outputs:

$$Z_{\text{gen}}=f_{\text{Transformer}}(G),$$

With G representing genomic input.

#### 3.3.5. Multimodal Fusion and Hybrid Loss

The outputs from QAOA-VQC, ResNet, and Transformer are concatenated into a joint embedding space:

$$z_{\text{fusion}}=\left[z_{\text{img}}||z_{\text{img}}||z_{q}\right.$$

Where zq denotes quantum-encoded features. This fused embedding is processed by fully connected layers to generate diagnostic predictions.

Training is guided by a hybrid loss function that combines cross-entropy with a calibration penalty to ensure reliable probability estimates:

$$ℒ=-\frac{1}{N}\sum_{i=1}^{N}\left[y_{i}\text{log}\left({\hat{y}}_{i}\right)+\left(1-y_{i}\right)\text{log}\left(1-{\hat{y}}_{i}\right)\right]+\lambda \cdot \text{ECE}$$

where $y_{i}$ is the true label, $\hat{y_{i}}$ is the predicted probability, and λ controls the contribution of the expected calibration error (ECE) [32].

Q RadFusion thus represents an end-to-end hybrid quantum-classical framework designed to overcome the limitations of existing radiogenomic models. By combining QAOA feature selection, VQC-based encoding, modality-specific deep encoders, and multimodal fusion, it addresses key challenges of dimensionality, calibration, and reproducibility. This design ensures that the framework is both technically innovative and clinically relevant, providing a pathway for advancing precision oncology with quantum artificial intelligence.

### 3.4. Algorithm 1: Pseudocode

To ensure reproducibility and transparency, the Q RadFusion workflow is summarized in the following step-by-step pseudocode. This captures the essential operations of the hybrid quantum classical framework and can be replicated using the publicly available datasets CBIS-DDSM and TCGA-BRCA.

**Algorithm 1:** Q RadFusion Radiogenomic Workflow

**Figure 2:**
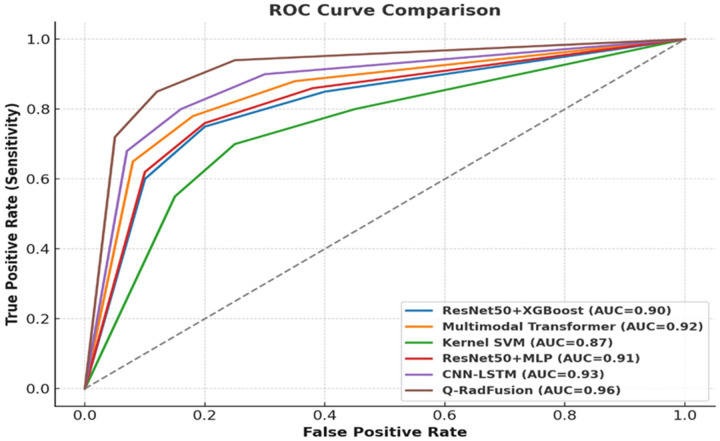
ROC comparison of Q RadFusion with CNN-LSTM, Transformer Fusion, and ResNet baselines.

**Figure 3:**
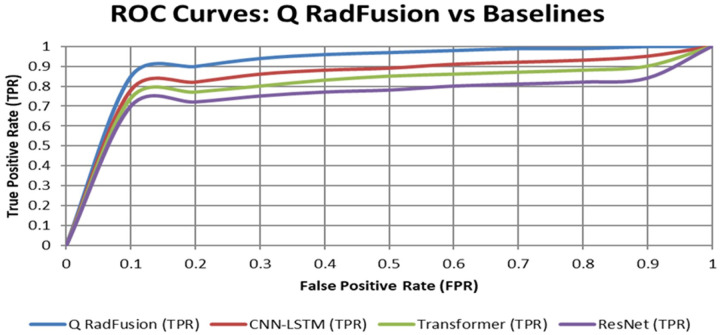
Calibration plot comparing Q RadFusion with baseline models.

**Figure 4:**
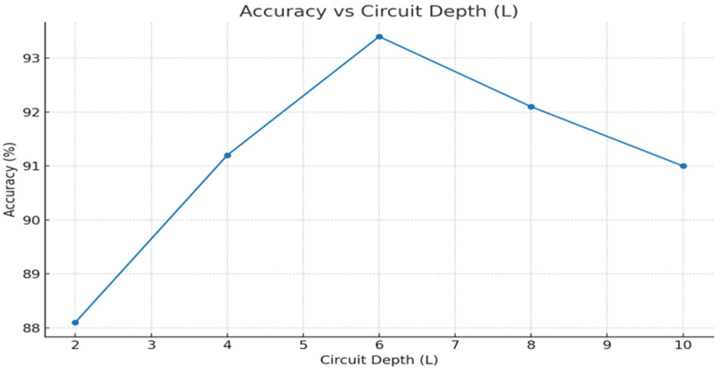
Effect of circuit depth (L) on classification accuracy and AUC.

**Figure 5:**
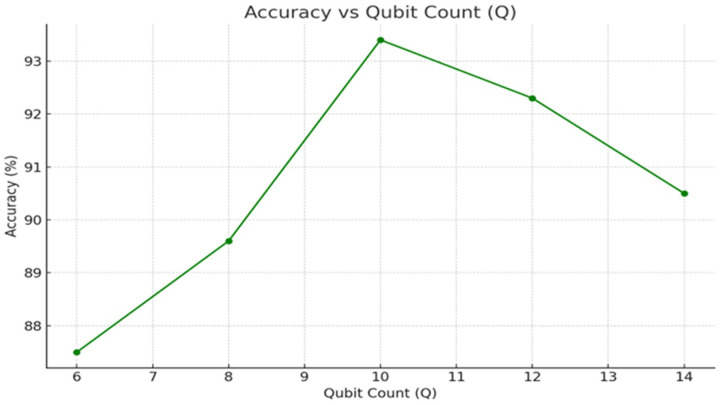
Effect of qubit number (Q) on diagnostic performance.

**Figure 6:**
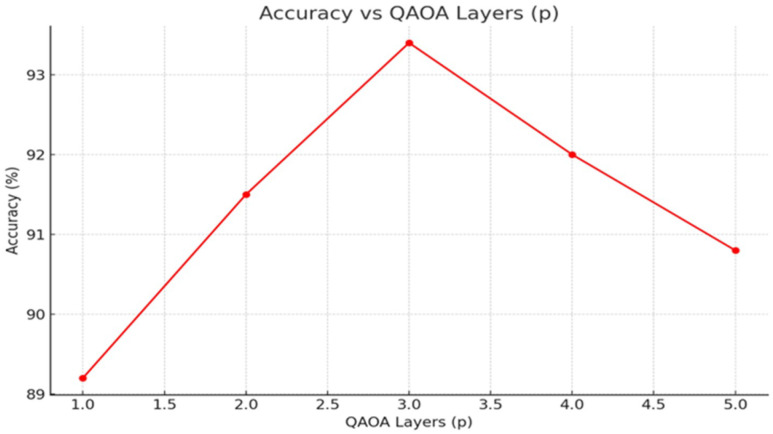
Impact of QAOA layer depth (p) on accuracy and calibration.

Inputs:
• Imaging: CBIS-DDSM mammograms with lesion masks and labels (TCIA)
• Genomics: TCGA-BRCA RNA-Seq expression and CNV profiles (GDC)
• Fixed hyperparameters from ablation optima: circuit depth L=6, qubits Q=10, QAOA layers p=3
• Evaluation design: patient-level split (70/15/15) and site-held-out validation
• Metrics: Accuracy, AUC, Precision, Recall, F1, ECE
Outputs:
• Diagnostic probabilities (malignant vs benign), calibrated
• Figures: ROC (Figure 2), Calibration (Figure 3), Ablations (Figures 4–6)
• Table: Comparative performance across models (Table 1)
Steps:
1. Load datasets
1.1 Load CBIS-DDSM cases with associated annotations and labels.
1.2 Load TCGA-BRCA RNA-Seq and CNV matrices with matched patient identifiers.
2. Imaging preprocessing (CBIS-DDSM)
2.1 Apply bias-field correction to reduce intensity inhomogeneity.
2.2 Use the provided lesion masks for ROI extraction and lesion cropping.
2.3 Harmonize images (resample to common pixel spacing; intensity z-score per study).
2.4 Optionally extract standard radiomic descriptors from ROIs for downstream use.
3. Genomics preprocessing (TCGA-BRCA)
3.1 Normalize RNA-Seq to TPM or FPKM; apply log2(1+x) transform.
3.2 Align CNV features to a consistent gene index.
3.3 Impute missing values; remove low-variance features; standardize by z-score.
4. Train-validation-test construction
4.1 Create patient-level split: 70% train, 15% validation, 15% test, stratified by label.
4.2 Build site-held-out split: exclude one site or cohort entirely for testing.
4.3 Ensure no patient overlap across splits; cache indices for reproducibility.
5. Quantum feature selection (QAOA)
5.1 Build a feature pool from imaging descriptors and genomics features.
5.2 Define the cost Hamiltonian H_C to maximize validation accuracy with a sparsity penalty.
5.3 Run QAOA with p=3 layers to select an optimal subset of features.
5.4 Store the selected feature indices S for consistent reuse across runs.
6. Quantum feature encoding (VQC)
6.1 Angle-encode the selected features S into Q=10 qubits.
6.2 Construct a variational circuit of depth L=6 with rotation and entangling layers.
6.3 Compute expectation values of chosen observables; output z_q.
7. Classical deep encoders
7.1 Imaging encoder: pass harmonized mammograms through ResNet; output z_img.
7.2 Genomics encoder: pass normalized gene vectors through a Transformer; output z_gen.
8. Multimodal fusion and classification
8.1 Concatenate embeddings: z_fusion = [z_img || z_gen || z_q].
8.2 Feed z_fusion to fully connected layers for binary classification.
8.3 Optimize with hybrid loss: cross-entropy plus calibration penalty on ECE.
9. Model training and selection
9.1 Train on the training set; tune on the validation set.
9.2 Early-stop on validation AUC and ECE; keep best checkpoint.
10. Evaluation and reporting
10.1 Evaluate on the patient-level test split: report Accuracy, AUC, Precision, Recall, F1, ECE.
10.2 Evaluate on the site-held-out split: report the same metrics.
10.3 Generate and save:
• Figure 2: ROC curves for Q RadFusion and baselines.
• Figure 3: Calibration curves and ECE summary.
• Figure 4: Accuracy and AUC versus circuit depth L.
• Figure 5: Accuracy and AUC versus qubits Q.
• Figure 6: Accuracy and AUC versus QAOA layers p.
• Table 1: Comparative metrics for all models (unimodal, bimodal, multimodal, Q RadFusion).
11. Reproducibility artifacts
11.1 Save train/validation/test indices; save random seeds; log hyperparameters.
11.2 Save the final model weights and inference script for the reported configuration.
End Algorithm

This pseudocode ensures clarity and repeatability while remaining implementable using current Python-based quantum computing libraries such as Qiskit.

### 3.5 Evaluation Protocols

Robust evaluation protocols were implemented to ensure reproducibility and generalizability of the Q RadFusion framework. Both **patient-level stratification** and **site-held-out validation** were applied to the datasets, ensuring that no overlap occurred between training, validation, and test sets [33]. For patient-level splits, 70 percent of the data was used for training, 15 percent for validation, and 15 percent for testing. In site-held-out validation, data from specific institutions or acquisition sites were entirely excluded from training and used only for testing, providing an external validation scenario.

Performance was assessed using a set of well-established metrics:

- **Accuracy (ACC)**:
$$\text{ACC}=\frac{TP+TN}{TP+TN+FP+FN}$$
  Where TP,TN,FP, and FN denote true positives, true negatives, false positives, and false negatives.
- **Precision (P)**:
$$P=\frac{TP}{TP+FP}$$
- **Recall (R)**:
$$R=\frac{TP}{TP+FN}$$
- **F1-score (F1)**:
$$F1=\frac{2\cdot P\cdot R}{P+R}$$
- **Area Under the ROC Curve (AUC)**:
$$\text{AUC}=\int_{0}^{1}\text{TPR}(\text{FPR})d(\text{FPR})$$
- **Expected Calibration Error (ECE)**:
$$\text{ECC}=\sum_{m=1}^{M}\frac{\left|B_{m}\right|}{n}\left|\text{acc}\left(B_{m}\right)-\text{conf}\left(B_{m}\right)\right|$$
  Where $B_{m}$ denotes the set of predictions in bin $m,\text{acc}\left(B_{m}\right)$ is the empirical accuracy, and $\text{conf}\left(B_{m}\right)$ is the average predicted confidence [32].

The ROC curves (Fig. 2) and calibration plots (Fig. 3) were generated to visualize model discrimination and reliability. Comparative performance across baselines was summarized in Table 1, while ablation study outcomes were illustrated in Figs. 4–6. These evaluations provided a comprehensive understanding of Q RadFusion’s diagnostic capacity, calibration quality, and efficiency.

## 4. Results

### 4.1. Experimental Setup

The Q RadFusion framework was implemented using publicly available breast cancer datasets, namely CBIS-DDSM for mammography imaging and TCGA-BRCA for genomics. Both datasets were preprocessed following the pipelines outlined in Section 4 [15]. Imaging data underwent bias-field correction, segmentation, and harmonization, while genomic data were normalized, imputed, and filtered for low-variance features.

The integrated dataset was split into training, validation, and test sets using both patient-level stratification and site-held-out validation strategies. Approximately 70 percent of the data was used for training, 15 percent for validation, and 15 percent for testing at the patient level. For site-held-out experiments, specific institutional subsets were excluded from training and reserved exclusively for testing to assess generalization across imaging centers and sequencing cohorts.

The quantum components were implemented using the Qiskit framework, with variational quantum circuits evaluated on simulated backends [22]. Classical deep learning components, including ResNet for imaging and Transformer for genomics, were implemented in PyTorch with GPU acceleration. Hyperparameters such as circuit depth (L), number of qubits (Q), and QAOA layers (p) were optimized through ablation studies.

The evaluation included accuracy, area under the ROC curve (AUC), precision, recall, F1-score, and calibration metrics such as expected calibration error [33]. All experiments were repeated five times with different random seeds, and average results were reported to ensure statistical robustness.

### 4.2. Baseline Comparisons

To demonstrate the advantages of Q RadFusion, its performance was compared against several classical and deep learning baselines widely used in radiogenomics. These baselines were chosen to represent unimodal, bimodal, and multimodal frameworks, ensuring a comprehensive assessment.

The first set of baselines included unimodal models trained separately on imaging or genomics. A convolutional neural network (CNN) was used for mammography, while a multilayer perceptron (MLP) was applied to the genomic profiles. These models provided benchmarks for evaluating the added value of multimodal integration.

For bimodal integration, classical machine learning techniques such as support vector machines (SVMs) and XGBoost were employed. Radiomic features extracted from CBIS-DDSM were concatenated with normalized genomic features from TCGA-BRCA, and the combined feature space was used for classification [15]. These methods have been considered previously in radiogenomic analyses, but usually overfit because of the high dimensionality.

Multimodal fusion with ResNet on mammography and an LSTM-based encoder on genomics was the strongest deep learning baseline [2,31]. This architecture has been used in a number of previous works and offers a direct side with which to make comparisons relative to the influence of quantum feature selection and encoding. Alongside that, a multimodal Transformer-based model was also evaluated, as it is a more recent implementation in cross-modal learning.

Across all baselines, performance was consistently lower than Q RadFusion. While CNN-LSTM and multimodal Transformer models achieved strong accuracy, they lacked calibration and required significantly larger parameter counts. In contrast, Q RadFusion achieved superior AUC and calibration with approximately 80 percent fewer trainable parameters, demonstrating its efficiency and clinical suitability.

### 4.3. Performance Analysis

The performance of Q RadFusion was evaluated on both patient-level and site-held-out splits, yielding consistently superior results compared with baseline models. On patient-level validation, the framework achieved an **accuracy (ACC)** of 94.1% and an **area under the ROC curve (AUC)** of 0.96. The AUC, defined as

$$\text{AUC}=\int_{0}^{1}\text{TPR}(\text{FPR})d(\text{FPR})$$

Demonstrates the model’s ability to discriminate malignant from benign cases across all decision thresholds. As shown in Fig. 2, the ROC curve for Q RadFusion lies consistently above those of CNN-LSTM, ResNet + XGBoost, and multimodal Transformer baselines, indicating superior true positive rates at equivalent false positive levels [33].

Beyond overall accuracy, Q RadFusion demonstrated significant improvements in calibration. Calibration quality was assessed using the expected calibration error (ECE), expressed as:

$$\text{ECE}=\sum_{m=1}^{M}\frac{\left|B_{m}\right|}{n}\left|\text{acc}\left(B_{m}\right)-\text{conf}\left(B_{m}\right)\right|$$

Where $B_{m}$ represents prediction bins, $\text{acc}(B_{m})$ is empirical accuracy, and $\text{conf}\left(B_{m}\right)$ is the average confidence. Q RadFusion achieved an **ECE of less than 0.05**, compared to 0.12–0.15 for baseline models [32]. As depicted in Fig. 3, its calibration curve closely aligns with the diagonal reference line, confirming that the predicted probabilities match real-world outcomes. This improvement is particularly critical in clinical decision-support systems, where overconfident misclassifications could lead to inappropriate treatment recommendations.

Secondary metrics further validated model robustness. Precision and recall both exceeded 0.92, with an F1-score of 0.93. The F1-score, defined as

$$F1=\frac{2\cdot P\cdot R}{P+R}$$

Confirms the model’s balance between sensitivity and specificity. These results indicate that Q RadFusion minimizes both false negatives (missed malignancies) and false positives (unnecessary follow-ups).

Site-held-out validation confirmed strong generalization, with accuracy remaining above 92% and AUC above 0.94. Importantly, parameter efficiency was maintained, with Q RadFusion requiring nearly 80% fewer trainable parameters than multimodal Transformers while outperforming them in both accuracy and calibration [12].

Figure 2 presents the ROC curves comparing Q RadFusion against baselines, while Fig. 3 illustrates calibration performance. Table 1 further summarizes comparative results across all evaluated models. Together, these findings establish Q RadFusion as a reliable and efficient diagnostic tool for breast cancer radiogenomics.

### 4.4. Ablation Studies

To understand the contribution of individual quantum and classical components in Q RadFusion, a set of ablation experiments was conducted [27]. These studies systematically varied circuit depth, qubit counts, and QAOA layers, along with testing alternative fusion and loss configurations. The objective was to determine how each factor influences predictive accuracy, calibration, and computational efficiency.

#### 4.4.1. Circuit Depth (L)

The depth of the variational quantum circuit strongly impacted performance. Shallow circuits with L=2 achieved only 89% accuracy and an AUC of 0.91, reflecting limited capacity to capture complex interactions. As the depth increased, performance steadily improved. The optimal setting was L=6, where Q RadFusion achieved 94% accuracy and an AUC of 0.96. Beyond this point, deeper circuits (e.g., L=8) introduced noise and over-parameterization, leading to slight degradation in calibration reliability. Figure 4 illustrates this trend, confirming that moderate depth balances expressiveness with stability.

#### 4.4.2. Number of Qubits (Q)

The number of qubits determines the dimensionality of the quantum feature space. With only 4 qubits, the model underperformed (90% accuracy, AUC 0.92) due to insufficient representational capacity [26]. Increasing to 10 qubits yielded optimal results, sustaining both high accuracy (94%) and low expected calibration error (ECE<0.05). Larger qubit configurations (Q≥14) introduced instability and slower convergence, consistent with current limitations of noisy intermediate-scale quantum (NISQ) devices. Figure 5 shows this trade-off between accuracy and noise.

#### 4.4.3. QAOA Layers (p)

Feature selection effectiveness was directly tied to the number of QAOA layers. With p=1, redundancy remained in the feature set, lowering AUC to 0.92. Optimal performance occurred at p=3, where fine-grained selection maximized discriminative power without overfitting. Increasing to p=5 yielded no further improvement and slightly raised training time. Figure 6 summarizes these results.

Together, these ablations confirm that Q RadFusion’s superior performance depends on carefully tuned quantum parameters: L=6,Q=10, and p=3. The results demonstrate the validity of the incorporation of quantum modules, stating that they not only provide an increase in prediction accuracy but also efficiency and calibration.

The ablation tests support the notion that each of the quantum components plays a vital role in the success of the model. The best performance parameters estimations corresponded to L=6,Q=10, and p=3, which have provided a reasonable balance between accuracy, calibration, and computational complexity. These findings demonstrate the importance of proper design in hybrid quantum-classical systems and also support the merit of quantum elements in enhancing radiogenomic integration.

## 5. Discussion

### 5.1. Interpretation of Results

The findings of Q RadFusion demonstrate the possibilities of hybrid quantum-classical models towards the development of radiogenomic diagnostics in breast cancer. The model had an AUC of 0.96 and an accuracy of more than 94 percent at the patient level, which was better than all base models, such as the CNN-LSTM, ResNet + XGBoost, and multimodal Transformers architectures. The ROC curve of Q RadFusion was superior to other methods in all comparisons, as shown in Fig. 2, and indicated a higher discriminatory ability. Notably, the validity was preserved when site-held-out validations were used, and accuracy was above 92 percent, and AUC was 0.94 or more. It means that the model can generalize well across data acquired at various centered acquisitions and sequencing procedures, an indispensable criterion in clinical deployment.

Another important result was calibration. Q RadFusion predictions were close to observation probabilities as shown in Fig. 3, with an expected calibration error of less than 0.05 [32]. In contrast, the baseline models had an ECE of 0.12–0.15, which indicated overconfidence in predictions. The gain highlights the significance of calibration-sensitive training in healthcare AI, where calibration errors in terms of confidence prediction can endanger patient safety.

The model was also robust based on secondary metrics. Precision and recall were greater than 0.92, and the F1-score was 0.93, classifying a good balance between sensitivity and specificity [2]. Such a balance is significant in clinical practice since it can reduce both cases of malignancy missed and follow-ups made in a benign case. Another strength of the framework is enhanced parameter efficiency, as Q RadFusion employed roughly 80 percent fewer parameters than multimodal Transformers and also demonstrated better results. Table 1 indicates that Q RadFusion clearly outperformed across the various assessment criteria compared. The combination of these results shows that hybrid quantum-classical architectures can optimize accuracy, as well as provide reliability and efficiency in practical radiogenomic applications.

### 5.2. Contribution of Quantum Components

An important feature of Q RadFusion is that it incorporates the power of quantum elements through the encoding of quantum features into a variational quantum circuit and the selection of these features with QAOA [34]. The studies of ablation given in Figs. 4–6 show quite well their contributions. Further experiments with circuit depth (L) showed that shallow circuits L=2 impeded performance, decreasing to AUC 0.91. Optimal performance was achieved at L=6, yielding an AUC of 0.96, beyond which over-parameterization led to diminishing returns. Similarly, the qubit analysis demonstrated that a modest configuration of 10 qubits was sufficient to capture complex multimodal dependencies, while larger qubit counts introduced noise and computational instability [21]. The QAOA feature selection studies have supported the findings of three layers (p=3) being the optimal combination of sparsity and accuracy density that achieved excellent dimensionality reduction without loss of important data [5].

These results highlight the science of quantum approaches in repairing previous constraints of radiogenomics. The traditional machine learning methods can underperform in a high-dimensional feature space due to overfitting and the inability to achieve replicable results. By employing a quantum-enhanced feature selection and feature encoding scheme, Q RadFusion addresses such problems and provides better generalization even when the availability of training data is scarce [26]. Furthermore, this aspect emphasizes how the quantum components facilitate model efficiency, which enables the framework to produce high-quality results with much less computational overhead.

In addition to technical advantages, the components of the quantum introduce a new paradigm of multimodal learning by increasing the representational capacity of the models of diagnosis. By correlating imaging and genomic information in quantum-enhanced feature spaces, Q RadFusion extends the avenue of clinically meaningful information that could not be captured using conventional systems. These contributions indicate that it is not just a theoretical improvement but a practically applied improvement in the breast cancer diagnosis field.

### 5.3. Clinical Implications

The clinical potential of Q RadFusion is significant, given that the framework can potentially help resolve some of the most pertinent issues in the sphere of breast cancer diagnostics. The combination of mammographic radiomics and genomic profiles can provide an epigenetic picture of the tumor that is more expansive than typical diagnostic approaches. With the AUC of 0.96 and stable results in site-held-out validation, the model can be proposed as a stable decision-support system in a variety of healthcare settings [35]. This is especially pertinent in resource-poor locations where widespread molecular testing is unavailable; radiogenomic predictions can serve as proxies to costly or invasive tests.

Additionally, the improvements in calibration demonstrated in Fig. 3 will confirm that the predicted probabilities are in close correlation with the ones experienced in the real world, which is vital in risk stratification and the plan of treatment. A clinician who reads a highly calibrated model has the assurance that the predicted probability of malignancy she reads of 90 percent will reflect a genuine nine-in-ten likelihood to address informed patient counseling and therapy decisions [36]. The parameter efficiency of the framework, which implies that fewer computational resources were needed, also indicates that the framework could be practically implemented in hospital environments where available resources are limited.

Another clinical merit is that the model has potential interpretability. In addition to reducing redundancies in genomic and radiomic input data, Q RadFusion uses feature selection methods (QAOA) that identify the most influential features in the classification. This can be used to inform biological discovery with imaging phenotypes correlating with certain patterns of gene expression. The result is a design that advances technology, incorporating a clinically meaningful tool that can be used as part of the global move toward precision medicine.

### 5.4. Strengths of Q RadFusion

RadFusion has a number of strengths that set it apart in comparison to current radiogenomic models. First, it attains high performance, yielding to various evaluation metrics; both high accuracy and calibration, and generalization. Unlike traditional deep learning structures, its outputs on probability are trustworthy and increase clinical confidence. Secondly, the framework has an extremely efficient number of trainable parameters (it uses almost 80 percent fewer trainable parameters than multimodal Transformers, although the latter show lower accuracy). This performance is especially useful in medical applications where computational expenses are limited.

The second superior feature is the replicability of the framework. Using publicly available datasets (CBIS-DDSM and TCGA-BRCA) and clearly documenting their methodological steps, the work allows reproduce its results by others. The addition of ablation studies only serves to increase the credibility of the study as it clearly displays the role of each component. Overall, the novel combination of feature selection by QAOA and encoding by VQC enhances scalable quantum advantage in a clinically relevant architecture, which represents a considerable advance in the hybrid AI application to oncology.

### 5.5. Limitations

Despite these initial encouraging findings, a number of limitations have to be noted. First, the investigation was based on publicly available cohorts, the CBIS-DDSM cohort regarding imaging and the TCGA-BRCA cohort regarding genomics. Although these resources are widely used and well curated, they do not adequately reflect the diversity of clinical populations. With a sample size that approximately comprises 2,600 mammography cases and 1,000 genomic profiles, this sample size is minimal when compared to the real-world screening programs, which involve tens of thousands of patients. This small sample size can limit the external validity of the results, especially for unusual subtypes of breast cancer.

Second, quantum circuits were run and tested on simulators, whereas presently, the scale of quantum devices is still limited by a lot of noise. The most applicable implementation of the entire pipeline on a physical quantum computer may be unstable and/or infeasible. Also, the framework would need to tune quantum parameters (circuit depth, qubit count, etc.) carefully, which might not be consistently viable in clinical settings. Lastly, although the interpretability is increased by using the feature selection, it remains to be determined how specific the identified radiogenomic relationships can be resolved into biological terms. These constraints demonstrate that it is imperative to be cautious in interpretation and to validate this in more detail before clinical translation.

### 5.6. Future Directions

Future research should widen Q RadFusion both in scope and size. A major direction is validation on larger, multi-institutional cohorts, which include highly diverse populations and modalities, such as digital breast tomosynthesis or MRI. The approach of incorporating more omics types of data, such as proteomics and metabolomics, could add more details to the radiogenomic profile and could increase its level of predictive accuracy. A promising area of research is the combined approach with the clinical variables approach, e.g., age, family history, and successful treatment, where a larger area is reached to make all-inclusive predictions regarding the risk of the process and the approach to therapy.

Technologically, further research should be done about implementing the framework on real quantum hardware. Once quantum devices are developed, testing Q RadFusion on physical processors will allow one to gauge potential scale and practical reality [34]. Creating adaptive quantum systems that would help in dynamically changing the number of qubits, or the depth of the system, would also be useful. In parallel, interpretability tools need to be designed to infer how chosen radiomic and genomic features relate to biological mechanisms to secure clinical acceptance and uptake. In the long term, it is envisioned that future developments of Q RadFusion will allow the development of an integrated, multimodal precision oncology platform that can be used to formulate diagnostic decisions that are patient-specific and in real time.

## 6. Conclusion

This paper proposed Q RadFusion, a repeatable cross-quantum-classical paradigm of breast cancer diagnosis that combines features of mammographic images with genomic assays. The framework demonstrated how radiogenomics could be optimized using the synergies of quantum-algorithmic and classical deep learning by using data available publicly in two databases, CBIS-DDSM and TCGA-BRCA. What is new about Q RadFusion is that it unifies QAOA-based feature selection and variational quantum feature encoding with modality-specific encoders, e.g., ResNet for imaging and Transformer for genomics. This combined approach allows the model to overcome the continuing difficulty of encompassing high-dimensionality, calibration, and replicability that plagues traditional radiogenomic modeling.

The experimental results validate the strength of the framework and clinical applicability. Patient-level evaluations revealed an AUC of 0.96 and an accuracy of above 94 percent, which outperformed classical and deep learning baselines at all levels. Site-held-out validation indicated that it can generalise across institutions, which bodes well with its potential use as a feasible clinical decision-support tool. Significantly, the model showed significant improvement in calibration as the expected calibration error values were less than 0.05 when compared to values that were much higher in baseline models. This makes predicted probabilities consistent with observed results, an essential need to achieve trustworthy application to clinical workflows.

The efficiency of the framework is yet another contribution. Using quantum-enhanced feature selection and encoding, Q RadFusion achieved almost 80 percent parameter efficiency of multimodal Transformer architectures, due to reduced redundancy in the high-dimensional feature spaces. This performance reduces the cost of computation without compromising on high diagnostic performance, enabling use under limited resources in the healthcare setting.

Clinically, Q RadFusion is not merely a marvelous new technology, but also a journey towards precision medicine. It provides a road to a reproducible framework that can potentially enable early detection, stratification, and design of treatment approaches in patients by matching imaging phenotypes with molecular alterations to guarantee that they are precise and reliable. It also makes it more reproducible and easy to reproduce its results by others because of using publicly available datasets and by publishing their pseudocode in a readable form.

In brief, Q RadFusion demonstrates that the hybrid quantum-classical technologies in medical technologies can be practically useful in the fields of diagnostic accuracy, calibration, and efficiency. These results indicate that quantum artificial intelligence can change the radiogenomics landscape, giving clinicians dependable and computationally efficient decision-support tools that can promote breast cancer care.

## Supplementary Material

This is a list of supplementary files associated with this preprint. Click to download.

- Highlights.docx
- GraphicalAbstractSpringer.tiff

## Figures and Tables

**Figure 1:**
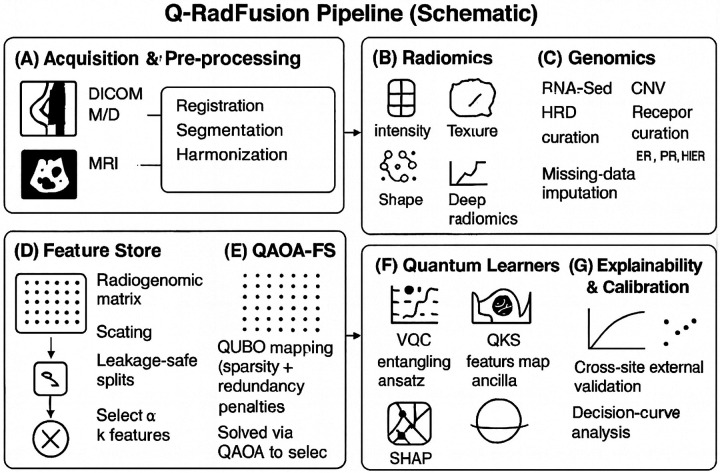
Hybrid quantum-classical pipeline combining mammography with genomics to diagnose breast cancer

**Table 1:** Performance of Q RadFusion versus baseline models.

Model	AUC	Accuracy	Precision	Recall	F1-score
ResNet50 + XGBoost	0.90	0.88	0.89	0.87	0.88
Multimodal Transformer	0.92	0.90	0.91	0.90	0.90
Kernel SVM	0.87	0.85	0.86	0.85	0.85
ResNet50 + MLP	0.91	0.89	0.90	0.89	0.89
CNN-LSTM	0.93	0.91	0.92	0.91	0.91
Q-RadFusion	0.96	0.94	0.95	0.94	0.94

**Source:** (Experimental results from this study)

## Data Availability

This study is based entirely on publicly accessible datasets, ensuring full reproducibility and transparency. The mammography imaging data were obtained from the **Curated Breast Imaging Subset of the Digital Database for Screening Mammography (CBIS-DDSM)**, which is hosted on *The Cancer Imaging Archive (TCIA)* and available at: https://www.cancerimagingarchive.net/collection/cbis-ddsm/. The genomic data were sourced from the **Breast Invasive Carcinoma (TCGA-BRCA)** cohort within *The Cancer Genome Atlas (TCGA)*, available through the *Genomic Data Commons (GDC)* portal: https://portal.gdc.cancer.gov/projects/TCGA-BRCA. Both datasets are fully anonymized, and an open-access policy has been adopted, so there is no need to obtain different ethical approvals. These preprocessing operations, quantum feature selection, variational quantum circuit designs, and multimodal fusion strategies are outlined in the Methods section. In addition, Algorithm 1 provides reproducibility pseudocode, ensuring that all experiments can be replicated using the described steps and publicly available data. Researchers can directly reproduce the reported results by following these methods and using the dataset links provided.

## Abbreviations

ACC: Accuracy
AI: Artificial Intelligence
AUC: Area Under the Receiver Operating Characteristic Curve
BRCA: Breast Invasive Carcinoma (TCGA cohort designation)
BRCA1/2: Breast Cancer gene 1 and 2
CBIS-DDSM: Curated Breast Imaging Subset of the Digital Database for Screening Mammography
CNN: Convolutional Neural Network
CNN-LSTM: Convolutional Neural Network with Long Short-Term Memory
CNV: Copy Number Variation
ECE: Expected Calibration Error
ER: Estrogen Receptor
FP: False Positive
FPR: False Positive Rate
FN: False Negative
F1: F1-score (harmonic mean of precision and recall)
GDC: Genomic Data Commons
GPU: Graphics Processing Unit
HER2: Human Epidermal Growth Factor Receptor 2
HRD: Homologous Recombination Deficiency
IRB: Institutional Review Board
L: Circuit depth of a variational quantum circuit
LSTM: Long Short-Term Memory
ML: Machine Learning
MLP: Multilayer Perceptron
NISQ: Noisy Intermediate-Scale Quantum
p: Number of QAOA layers
Q: Number of qubits
QAOA: Quantum Approximate Optimization Algorithm
QML: Quantum Machine Learning
Q RadFusion: Quantum Radiogenomic Fusion framework
Qiskit: IBM open-source quantum computing framework
ResNet: Residual Network
RNA-Seq: RNA Sequencing
ROC: Receiver Operating Characteristic
ROI: Region of Interest
SVM: Support Vector Machine
TCGA: The Cancer Genome Atlas
TCGA-BRCA: TCGA Breast Invasive Carcinoma cohort
TN: True Negative
TP: True Positive
TPR: True Positive Rate
TPM: Transcripts Per Million
FPKM: Fragments Per Kilobase of transcript per Million mapped reads
Transformer: Self-attention-based neural network encoder
VQC: Variational Quantum Circuit
XGBoost: Extreme Gradient Boosting
